# ‘Bee Hotels’ as Tools for Native Pollinator Conservation: A Premature Verdict?

**DOI:** 10.1371/journal.pone.0122126

**Published:** 2015-03-18

**Authors:** J. Scott MacIvor, Laurence Packer

**Affiliations:** Biology Department, York University, Toronto, Ontario, Canada; Universidade de São Paulo, Faculdade de Filosofia Ciências e Letras de Ribeirão Preto, BRAZIL

## Abstract

Society is increasingly concerned with declining wild bee populations. Although most bees nest in the ground, considerable effort has centered on installing ‘bee hotels’—also known as nest boxes or trap nests—which artificially aggregate nest sites of above ground nesting bees. Campaigns to ‘save the bees’ often promote these devices despite the absence of data indicating they have a positive effect. From a survey of almost 600 bee hotels set up over a period of three years in Toronto, Canada, introduced bees nested at 32.9% of sites and represented 24.6% of more than 27,000 total bees and wasps recorded (47.1% of all bees recorded). Native bees were parasitized more than introduced bees and females of introduced bee species provisioned nests with significantly more female larva each year. Native wasps were significantly more abundant than both native and introduced bees and occupied almost 3/4 of all bee hotels each year; further, introduced wasps were the only group to significantly increase in relative abundance year over year. More research is needed to elucidate the potential pitfalls and benefits of using bee hotels in the conservation and population dynamics of wild native bees.

## Introduction

**Fig 1:**
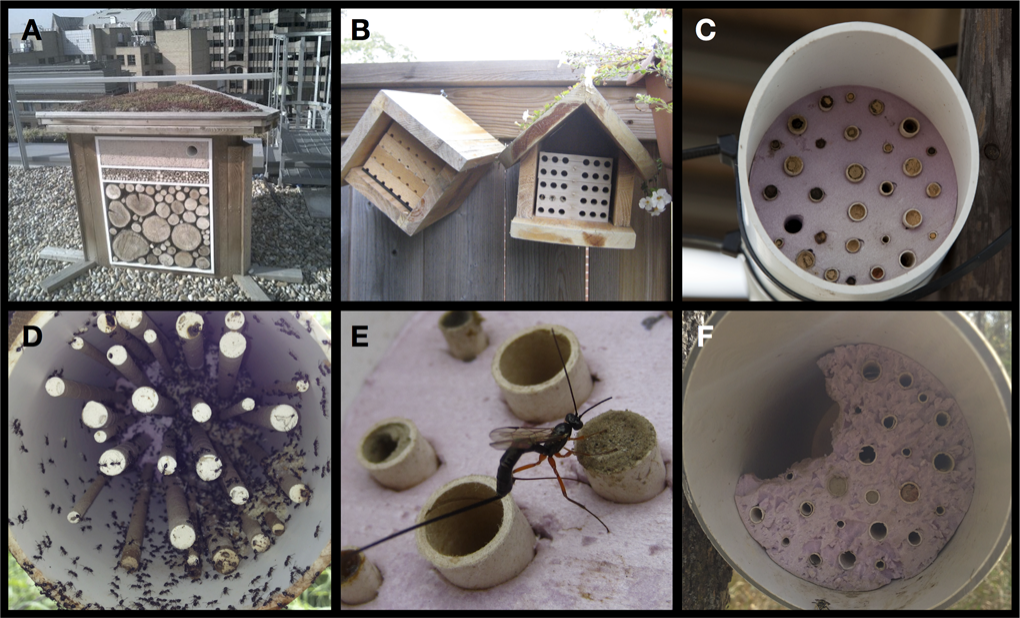
Bee hotels. A. Bee hotel on a rooftop in London, UK (Photo: Thierry Spiess). B. Cartridge-style hotels made by bundling wood (left) or plastic (right) cartridges having drill holes along one edge for opening, inspecting and cleaning. C. Bee hotel having different nesting tube widths made of cardboard and enclosed in a PVC pipe for protection (Photo: Ed Snodgrass). D. Ant colony (*Tetramorium caespitum*) that took over an unmaintained bee hotel. E. An ichneumonid wasp parasitizing *Osmia* sp. through a cardboard nesting tube. F. Damage to the faceplate and nesting tubes in a bee hotel by an unknown bird.

Bees and the pollination services they provide are in decline as a result of various anthropogenic activities that undermine bee foraging and nesting [1,2,3,4]. Concern for bees among the general public has led to increases in the numbers of novice beekeepers in urban centers [5] and augmentation of habitat for bees including the addition of both food (bee-friendly plants) [6,7] and nest sites (bee hotels) [8]. The marketing of bee hotels to promote pollination and wild pollinator conservation is widespread and expanding, at least in North America and Europe [9]. These structures, also known as trap-nests or nest boxes [10], use some bee’s preferences for nesting in above-ground cavities as arise naturally in a variety of settings such as pithy stems and beetle burrows in wood [11,12]. Bee hotels are usually made from bundled plant stems, paper-based tubes, or holes drilled in wood or molded in plastic; in all cases they artificially aggregate nesting sites above densities naturally available for cavity-nesting bees [10] (Fig. 1A-C).

Bee hotel development began in the 1950s when paper straws and wooden blocks with holes drilled into them were experimentally set out to house the alfalfa leaf cutter bee [*Megachile rotundata* (Fabricius)] in transportable and stackable containers [10]. At that time, farmers from Utah to Saskatchewan were encouraging this exotic species to nest in holes they had drilled into the sides of their own buildings [13]. Over the ensuing decades there has been an increasing diversity of designs available for purchase as ready-mades or through DIY instructions (e.g. http://www.xerces.org/). In agricultural settings, a variety of mason bees, in addition to the alfalfa leafcutter bee, have been managed successfully using bee hotels [13,14,15]. These easily manipulated structures have also been used for ecological research [16,17,18,19]. Promotion of bee hotels in urban gardening as a means of supporting native pollinators is a more recent phenomenon. Here we investigate whether they do indeed support native pollinators rather than introduced ones or other organisms entirely. Specifically, we test the following hypotheses.

1. Compared to native bee species, introduced ones are more common in bee hotels. Introduced species often exhibit greater flexibility in habitat requirements [20,21], allowing them to colonize new environments; bee hotels may constitute such a novel environment.
2. Wasps (such as many solitary Vespidae) that seek out the same nesting cavities will be more common than native bees in bee hotels because wasps use widely available nesting materials to partition their nests (e.g. mud and grass) whereas bees use more site specific materials (e.g. tree resins and leaves of certain plants) [10,11,22].
3. Introduced species will be more common in bee hotels located in areas that are most heavily anthropogenically-modified. This is expected because recent studies that investigate urban insect diversity find introduced species to be the dominant taxa [23,24].
4. Compared to native species, introduced ones will have decreased rates of parasitism. This is a test of whether the enemy release hypothesis [25] applies to bees that nest in bee hotels. In bee hotels, parasitism is greater compared with natural nesting sites [26] in part because aggregated nests create an easier search target for parasites [27]. This may exacerbate the differences in parasitism rates between native and exotic species.

If hypotheses 1, 3 and 4 were to be supported we could suggest the following two additional hypotheses:

5 Introduced bees will show a greater increase in bee hotel use over time from year to year.
6 Introduced bees will exhibit greater population increase (expressed as number of females per nest tube) than native species.

We test these hypotheses with 200 bee hotels set up annually for each of three years within the city of Toronto, Canada. We test the first five hypotheses using all bees and wasps detected; the fifth and sixth were explored using two congeneric pairs of the commonest species found, in each case one member of the pair was introduced, the other native.

## Methods

From May to October 2011–2013, 200 bee hotels were set up each year throughout the Toronto area (each bee hotel representing one ‘site’) to survey above ground nesting bees [19]. The majority of sites were sampled all three years (73.7%), 16.9% were sampled over two years, and 9.4% were sampled in just one year. The bee hotels were made from 10cm-diameter, 28cm long white PVC piping, with a circular faceplate made of insulation board into which 30 cardboard nesting tubes (10 of each of three tube diameters; 3.4mm, 5.5mm, 7.6mm; all 15cm in length) could be mounted (Fig. 1; see [19] for more detail). A bee that uses the hotel enters a suitable cardboard tube (the one that best fits her body dimensions) and constructs brood cells in a series from the back of the tube to the front [10,11,28]. Bee hotels were set up individually at sites at least 250m apart, in four different urban green space types: community gardens, residential gardens, city parks, and building rooftops. Permission was granted to set up at each site after meeting with individual site managers or homeowners to discuss the research.

At the end of each field season, the bee hotels were collected, each cardboard tube opened and each brood cell removed, individually labeled and placed in storage to overwinter at 4°C. In April of the following year brood cells were moved to a sealed incubation chamber kept at 26°C and 60% humidity until adult emergence. They were then sexed and identified to species, permitting categorization of each individual as native or introduced to the study region [29,30,31]. All bees and wasps are stored at the Packer Collection at York University (PCYU). Over all sites and years, colonization (determined as presence in a bee hotel) and relative abundance (the proportion of all brood cells that were of the focal species per bee hotel) were compared between native and introduced bees (NB vs IB), native and introduced wasps (NW vs. IW), as well as among all four groups using linear regression analysis (GLM) (α = 0.05) with a Tukey post hoc analysis in SPSS v21 (all analyses described hereafter used this program). Colonization and relative abundance of native bees (NB) were also compared with all potential competitors of native bees for nesting opportunities in bee hotels (introduced bees and introduced and native wasps grouped together; hereafter referred to as “AO”, as in “all others”) using a paired t-test. The same GLM test was used to determine whether colonization or relative abundance between native and introduced bees and wasps differed by site type.

The total number of parasites attacking bee and wasp brood were recorded by site, and the parasites reared and identified as accurately as possible using standard morphological approaches combined with DNA barcoding [32]. The total parasitism rate combining all brood cells over all three years were compared separately as before between native and introduced bees and wasps using GLM analysis with Tukey post hoc testing to distinguish between different bee and wasp groups. Although the bees and wasps we sampled were not released back to the site from where they were collected, to examine patterns in use over time, the abundance of each group per site were independently examined over the three years using a repeated measures ANOVA with data from the first year of sampling acting as a baseline for comparison. This was completed only for the sites sampled in all three years (N = 147).

We compared the sex ratio, as well as an estimate of the rate of increase in population size of the four most common bee species over the three-year study period. The most common bees were two native [*Osmia pumila* Cresson, *Megachile campanulae* (Robertson)] and two introduced species [*O*. *caerulescens* (L.) and *M*. *rotundata*] (Table 1). The estimate of population increase for each species was determined by comparing the number of female offspring provisioned by nesting females in 30 individual nests of the same nesting tube width dimension (1 per site; 10 sites selected randomly among those colonized by the species in all three years). The sex ratio (recorded as the proportion of females per nest) and the estimate of population increase over the study period were independently compared for two pairs of bee species using GLM testing and *post hoc* analysis.

**Table 1:** List of all bee species recorded in the study area per year (Y: yes; N: no).

Family	Genus	Species	2011	2012	2013
Apidae	*Anthophora*	*terminalis* Cresson	N	N	Y
Megachildae	*Megachile*	*brevis* Say	N	Y	N
		*campanulae* (Robertson)	Y	Y	Y
		***centuncularis*** (Linnaeus)*	Y	Y	Y
		*frigida* Smith	N	Y	Y
		*inermis* Provancher	Y	N	N
		*mendica* Cresson	Y	Y	N
		*pugnata* Say	Y	Y	Y
		*relativa* Cresson	Y	Y	Y
		***rotundata*** Fabricius	Y	Y	Y
		***sculpturalis*** Smith	N	N	Y
	*Heriades*	*carinata* Cresson	Y	Y	Y
		*variolosa* (Cresson)	Y	N	N
	*Chelostoma*	***campanularum*** (Kirby)	N	Y	Y
		***rapunculi*** (Lepeletier)	Y	N	Y
	*Hoplitis*	*producta* (Cresson)	Y	Y	Y
		*spoliata* (Provancher)	Y	Y	N
		*truncata* (Cresson)	N	N	Y
	*Osmia*	*pumila* Cresson	Y	Y	Y
		***caerulescens*** (Linnaeus)	Y	Y	Y
		*lignaria* Say	Y	Y	Y
		*atriventris* Cresson	N	Y	N
	*Anthidium*	***manicatum*** (Linnaeus)	Y	N	Y
	*Coelioxys*	*alternata* Say^+^	Y	N	N
		*moesta* Cresson ^+^	N	Y	N
		*sayi* Robertson ^+^	Y	Y	Y
	*Stelis*	*lateralis* Cresson ^+^	Y	Y	N
		*vernalis* Mitchell ^+^	N	Y	N
Colletidae	*Hylaeus*	*affinis* Smith	Y	Y	Y
		*annulatus* (Linnaeus)	Y	Y	Y
		***hyalinatus*** Smith	N	Y	Y
		***leptocephalus*** Morawitz	N	Y	Y
		*mesillae* (Cockerell)	Y	N	Y
		*modestus* Say	Y	Y	Y
		***punctatus*** Brullé	Y	Y	N
		*verticalis* (Cresson)	N	Y	N

Bolded species are introduced to the study region. Missing introduced cavity-nesting bees known from the region included: *Anthidium oblongatum* (Illiger), *Hoplitis anthocopoides* (Schenck), and *Megachile ericetorum* Mitchell.

* *M*. *centuncularis* status is not clear, with Giles and Ascher (2006) denoting the species as introduced.

^+^ denotes species that are cleptoparasites.

## Results

Of 600 bee hotel/years set up, data were obtained from 574 (186 were recovered in 2011, 194 in 2012 and 194 in 2013). We found a total 27,275 individuals including 31 species of pollinating bees (comprising 52% of all cavity-nesting bee species known from the area [29]) and an additional five cleptoparasitic bee species (36 bee species total) (Table 1). Ten of the species we found were not native to the region, representing 76.9% of the known introduced cavity-nesting bee fauna in southern Ontario (Table 1). The offspring generations of two introduced species: *O*. *caerulescens* and *M*. *rotundata* were particularly common; representing 20.7% and 15.4% respectively of the total number of bees reared over the entire study period.

**Fig 2:**
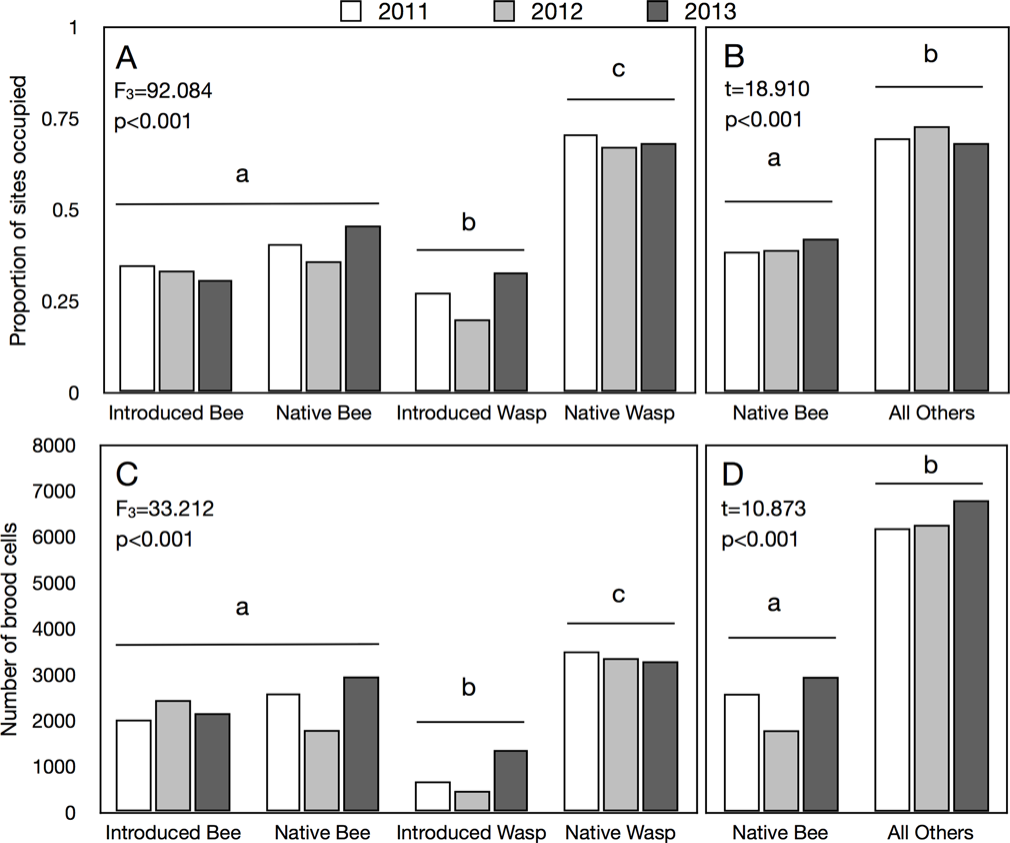
Presence and abundance of bees and wasps over all sampling years. A. The number of sites occupied by native bees (NB), introduced bees (IB), native wasps (NW) and introduced wasps (IW) over three years at over 600 bee hotels set up through out the city of Toronto and the surrounding region. B. Comparison of the number of sites occupied by NB and the other groups competing for nesting space combined (AO). C. The total number of brood cells produced in bee hotels per year by native and introduced bees and wasps, and D. shows a comparison between native bees and the remaining groups combined. Lower-case lettering indicates significant differences and in all graphs hereafter.

There was no significant difference between native and introduced bees in the number of sites occupied (Fig. 2A), with introduced bees nesting in bee hotels at an average of 32.9% of sites per year (native bees: 39.8%). Native bees colonized significantly fewer sites than did all other groups combined (AO: 70.5%) (Fig. 2B).

There was no significant difference in the relative abundance of introduced and native bees reared from bee hotels (Fig. 2C); introduced bees represented 47.1% of the total number of bees reared (56.9% for native bees), and 24.6% of all bees and wasps reared (27.6% for native bees). However, the relative abundance of native bees was significantly less (t = 9.239, p<0.001) than that of all competing groups combined (AO: 72.4%) (Fig. 2D). Native wasps were significantly more abundant than any other group (Fig. 2A; F_3_ = 20.46, p<0.001) and comprised 37.8% of all bees and wasps reared from bee hotels.

**Fig 3:**
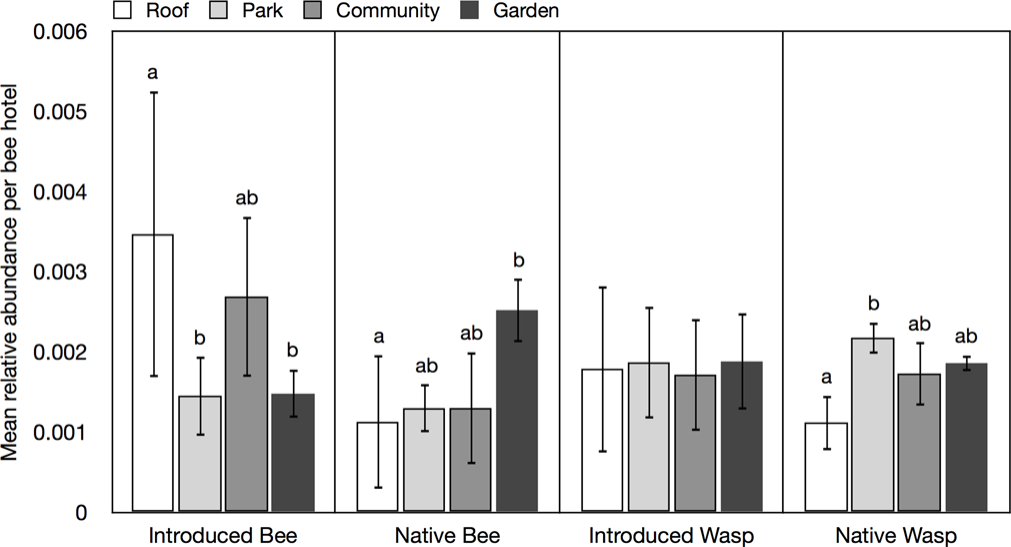
Mean relative abundance of bees and wasps at all site types over all years. Lower-case lettering indicates significant differences.

**Fig 4:**
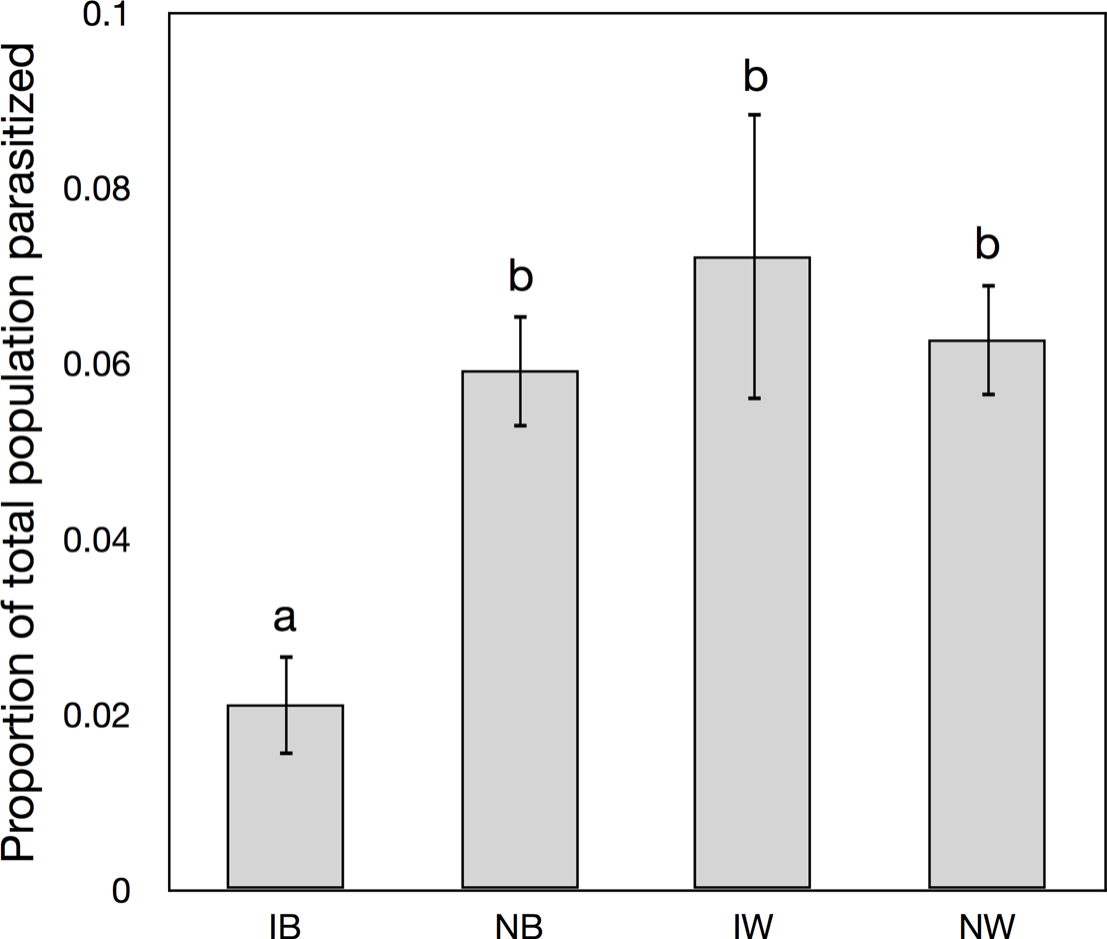
The proportion of parasitized brood cells from all sites and years combined.

The type of urban green space was a significant determinant of the abundance of native bees (F_3_ = 5.369, p = 0.001, greatest in residential gardens), introduced bees (F_3_ = 4.511, p = 0.004, greatest on rooftops, and to a lesser extent community gardens) and native wasps (F_3_ = 5.880, p = 0.006, greatest in urban parks), but not of introduced wasps (Fig. 3). Significantly more native bees were parasitized compared to introduced bees (t = 13.904, p<0.001) (Fig. 4), although parasitism rates did not differ between introduced and native wasps.

Repeated measures analysis showed that there was no significant change in relative abundance of native or introduced bees or native wasps year-over-year; however, there was a significant increase in introduced wasps (F_3_ = 6.555, p<0.001) (Fig. 2C).

**Fig 5:**
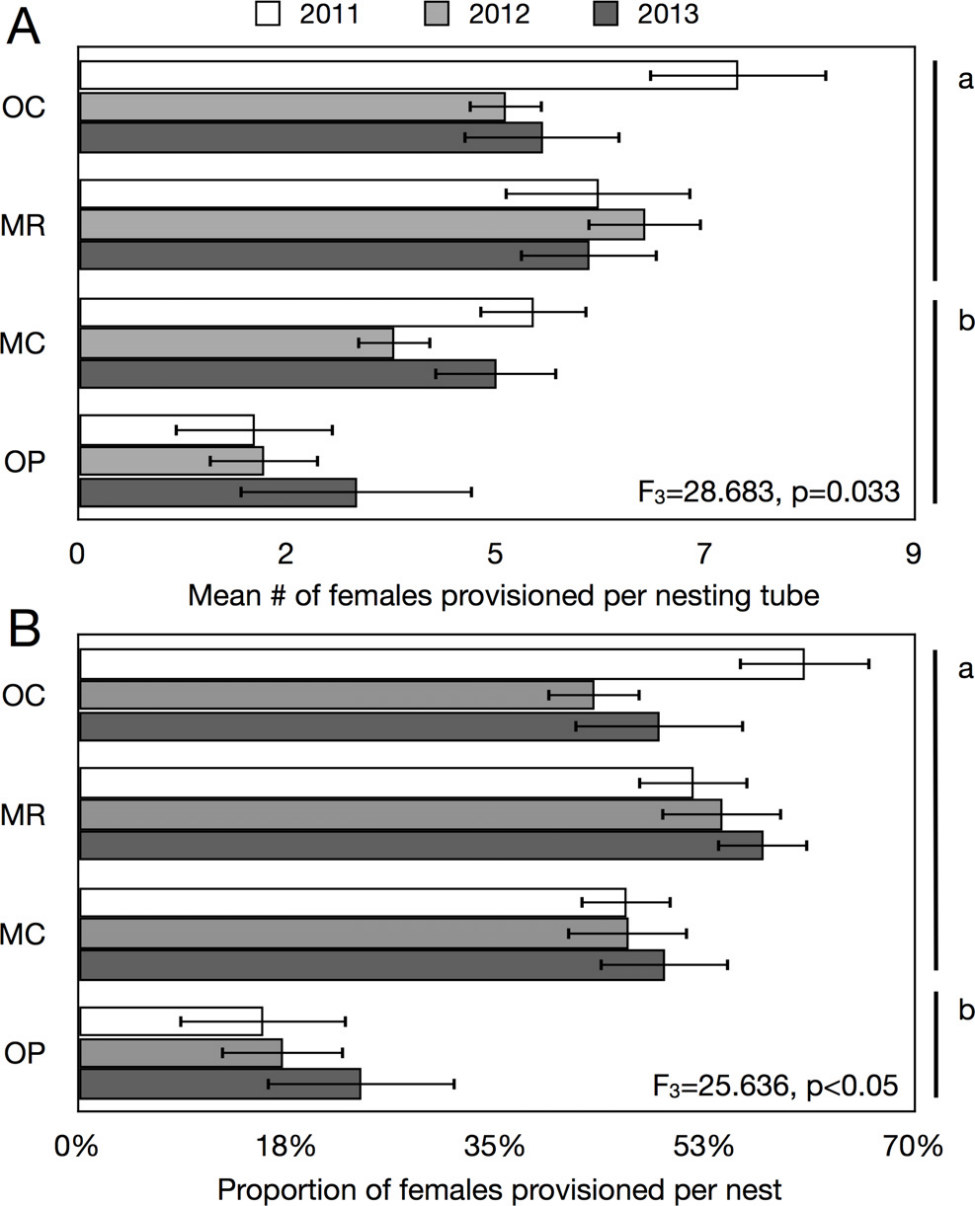
Number of females provisioned in nests by the most common native and introduced bees. A. Differences in the mean number of females in nests of four bee species [two introduced: *Osmia caerulescens* (OC), *Megachile rotundata* (MR); and two natives: *Osmia pumila* (OP), *Megachile campanulae* (MC)]. B. Estimates of the rate of population increase in those same bees as determined by the number of female offspring provisioned per individual nesting female over three years. Lower-case lettering reflects significant differences.

Finally, the sex ratio as determined by the number of females provisioned per preferred nesting tube width in the two pairs of native and introduced bees species was significantly more skewed towards females in introduced than native bees (F_3_ = 28.683, p = 0.033) (Fig. 5A). This trend was driven by one native, *O*. *pumila*, which provisioned, on average, half as many females as the other native (*M*. *campanulae*) per brood cell. *Osmia pumila* was the only bee among the four to prefer the 3.4mm nesting tube width (73.1% of all nest tubes occupied and 77.1% of all brood produced). The average number of female offspring provisioned per female did not change significantly for any of the four species over the three years of study (F_3_ = 0.738, p = 0.481). However the estimate of the rate of population increase differed significantly among species with both introduced bees (*M*. *rotundata*, *O*. *caerulescens*) and one native (*M*. *campanulae*) provisioning significantly more female offspring per nesting female compared to native *O*. *pumila* (F_3_ = 25.636, p<0.001) (Fig. 5B).

## Discussion

We investigated the relative use of bee hotels by native and introduced bees and wasps to assess the potential of these novel habitat augmentation schemes for increasing populations of native bees. Several lines of evidence suggested that native bees performed comparatively poorly.

First, although there was no difference in the abundance or colonization of bee hotels by introduced and native bees, native bees were in the minority, representing 27.7% of all bees and wasps reared (AO + NB). Thus, our hypothesis that introduced bees would use the hotels more often than native bees was rejected. This result was similar to that found in a study in California where native bees or wasps never amounted to more than 25% of bee hotel occupants over two years [21]. Grouping all potential competitors of native bees for nesting opportunities in bee hotels (AO), we found that their colonization rate and abundance was greater than that of native bees. Native wasps were significantly more abundant than native and introduced bees, and so our second hypothesis, that wasps could outcompete bees for these nesting structures was supported. Our third hypothesis—that site type, as determined by the type of urban green space where the bee hotel was installed—would influence the relative abundance of native bees was supported. Bee hotels in residential gardens had significantly more native bees (e.g. [33]) while more anthropogenically-modified sites (e.g. vegetated rooftops) supported significantly higher numbers of introduced bees (Fig. 3).

Our fourth hypothesis was that introduced bees would be parasitized less often than native bees. This pattern was evident when all years were combined (Fig. 4) and so we accepted our fourth hypothesis. From the repeated measures analysis and using the first year of abundance data per sites as a baseline for comparison, no significant difference in changes in abundance was evident from year to year for native or introduced bees, and we rejected our fifth hypothesis.

Our sixth hypothesis that introduced bees would exhibit greater population increase than native bees was partially supported. Our most common native bee, *O*. *pumila*, provisioned significantly fewer females per nest than did either of our two introduced bees (Fig. 5). *Osmia pumila* preferred smaller diameter nesting tubes than did our other three species and because males are smaller than females use of smaller diameter tubes is expected to result in a more male-biased sex ratio. For example, an increasingly male biased sex ratio was reported for *Osmia lignaria* in smaller sized nesting tube diameters [34]. To check whether reduced female production by *O*. *pumila* might have been an artefact of tunnel width preferences we looked at its sex ratio in tubes of both diameters. In 3.4mm tubes, 7.9% of the brood was female whereas in 5.5mm tubes 58.6% were females giving a total of 19.5% female overall in the population of *O*. *pumila*. The other three species preferred to nest in the 5.5mm nesting tubes (*M*. *campanulae* = 81.3% of all brood reared, *M*. *rotundata* = 60.2%, *O*. *caerulescens* = 77.8%).

Altogether, our study findings show that bee hotels appear to differentially augment populations of wasps rather than those of native bees, and introduced bees outperform at least some native bee species in some population parameters in bee hotels and in some urban green space types. These results highlight a need for increased study of bee hotels and their associated impact upon bee biodiversity and pollination in the urban setting.

One reason bee hotels are promoted is their potential for augmenting pollination of native plants [35] and/or crops [36]. However, introduced pollinators, which in this study represented almost half of all bees reared, are often the dominant or sole pollinator(s) of introduced plants [37,38,39], whereas, native bees prefer native plants to alien ones [40].

At their worst, bee hotels may act as population sinks for bees through facilitating the increase of parasites, predators (e.g. Fig. 1D-F), and diseases as a result of functional responses to unnaturally high nest densities and nesting site entrances set up in two-dimensions rather than in the more three dimensional arrangement found in nature (e.g. erect plant stems, decaying logs) [26]. Bee hotels may be designed to encourage different bee species by varying nesting tube/hole width or length, but encouraging different bee species to co-aggregate in a bee hotel might inadvertently increase opportunity for parasites to attack related species: developing novel hosts or affect more susceptible species [41]. Although there has been little discussion of parasite loads obtained with different bee hotel designs [11] in all cases where nesting sites and nesting bees are aggregated, the chance of parasites finding and attacking nests is increased [27]. Some bee hotels have thin-walled nest tubes that facilitate parasite transfer within the hotel, even by parasitic insects with short ovipositors such as generalist *Monodontomerus* wasps [42]. This can result in mortality of entire hotel contents [11]. The relative influence of host aggregation was not examined in this study, however we did find a significant increase in the total number of parasites attacking native bees compared to introduced ones. These findings might have resulted from enemy release among introduced bees, which were free of specialist parasites that attack them in their native ranges [25].

Given bee hotels could have a negative or a positive impact on their target organisms, an obvious question is: How can designs be modified to promote the desired outcomes? Finding answers to this would involve increased research on the parameters that vary among different bee hotel designs and their relative success at promoting native bees. This could include studies that manipulate the number, positioning/location, and materials used in bee hotel construction. The impact of maintenance might include replacement of completed nesting tubes with new unoccupied ones to reduce within-season competition for nest sites. Matching the length or especially the width [11,13] of nesting tubes in bee hotels to that of preferred plant stems and beetle-bored holes in wood could reveal parameters that increase attractiveness to specific native bees, reduce rates of parasitism, and/or increase the number of females provisioned per nest (e.g. [34,43]). For example, a nest tube diameter between 3.4mm and 5.5mm would seem to be necessary for population increase in *O*. *pumila*.

### “Bee-washing”: A Call for Research

We advocate for due diligence on the part of retailers and promoters of bee hotels to avoid “bee-washing”; that is, green-washing [44] as applied to potentially misleading claims for augmentation of native and wild bee populations. To ensure “bee-washing” is minimized, it is imperative that more research be performed on the design and effectiveness of bee hotels. Bee hotels are useful for ecological and behavioral studies, outreach in citizen science and pollinator education campaigns. Sampling with them can even reflect the diversity of the larger bee community (e.g. including bees that nest in the ground [45]). However the magnitude of potential pitfalls noted above needs to be assessed through continued study, especially of the impact of hotels on native bee population dynamics. Such work would also provide detailed data on the pollen and nesting resources used, parasite associations, sex ratios, and behaviors.

Comparing nesting success in bee hotels with that at naturally occurring nesting sites [46,47] could improve the effectiveness of this management tool and permit its integration into landscape planning practices targeting conservation. Specifically, through better designs modeled after natural conditions both in materials used and details of positioning in the environment. At present, some bee hotels marketed for consumer use may act as sinks for their target organisms through provision of entirely inappropriate edaphic conditions as a result of the materials used. For example, some designs are simply holes drilled (or molded) into solid plastic blocks. It seems highly improbable that these designs will provide the same moisture balance as occurs in nature and increased moisture retention likely leads to increased brood mortality due to mold, which can represent a large proportion of total brood mortality [48]. Set up and orientation could also be linked to attractiveness to native bees, especially versus wasps (e.g. [49]); for example, solitary wasps can be more prevalent in bee hotels placed in shaded conditions [22]. These wasps may provide important services to urban gardeners as predators of pests [50], but compete with bees for nesting space in bee hotels. This suggests the need for a deeper understanding of the relative importance of the pollination augmentation versus pest control potential of bee hotels. In sum, we advocate for more research and increased responsibility on the part of retailers and advocates of bee hotels so that these structures are designed and managed to minimize negative effects and become truly useful tools for conservation biologists and conservation-minded citizens.
